# 

**Published:** 1913-11

**Authors:** 


### CONTENTS.

ORIGINAL COMMUNICATIONS—

Surgical Kidney, by A. L. Fowler, M. D., Atlanta, Ga.. .335

A Case of Unilateral Pyonephrosis of Hematogenous Origin Treated by Nephrotomy, by R. M. Harbin, M. D., Rome, Ga. .....344

Refuse Hemmorrhage from the Deep Epigastric Artery, Ten Days After an Appendix Operation Through the Rectus Muscle, by Gaston Torrance, M. D., Birmingham, Ala. .....346

Physicians' Study Travels .....346

The American Society for Physicians' Study Travels....348

EDITORIAL .....349

SELECTIONS AND ABSTRACTS .....354

Regular Meeting Fulton County Medical Society, October 16, 1913, Reported by R. R. Daly, M. D..........372

BOOK REVIEWS .....381

